# Bacterial group I introns: mobile RNA catalysts

**DOI:** 10.1186/1759-8753-5-8

**Published:** 2014-03-10

**Authors:** Georg Hausner, Mohamed Hafez, David R Edgell

**Affiliations:** 1 Department of Microbiology, University of Manitoba, Winnipeg, MB R3T 2 N2, Canada; 2 Department of Biochemistry, Faculty of Medicine, University of Montreal, Montréal, QC H3C 3 J7, Canada; 3 Department of Botany, Faculty of Science, Suez University, Suez, Egypt; 4 Department of Biochemistry, Schulich School of Medicine and Dentistry, Western University, London, ON N6A 5C1, Canada

**Keywords:** Evolution, Group I introns, Intron splicing, Intron mobility, Homing endonuclease genes, IStrons

## Abstract

Group I introns are intervening sequences that have invaded tRNA, rRNA and protein coding genes in bacteria and their phages. The ability of group I introns to self-splice from their host transcripts, by acting as ribozymes, potentially renders their insertion into genes phenotypically neutral. Some group I introns are mobile genetic elements due to encoded homing endonuclease genes that function in DNA-based mobility pathways to promote spread to intronless alleles. Group I introns have a limited distribution among bacteria and the current assumption is that they are benign selfish elements, although some introns and homing endonucleases are a source of genetic novelty as they have been co-opted by host genomes to provide regulatory functions. Questions regarding the origin and maintenance of group I introns among the bacteria and phages are also addressed.

## Introduction

Group I introns are structured self-splicing introns that in part persist in genomes by minimizing the impact of their insertion into host genes. This is accomplished by autocatalyzing their removal (splicing) from primary transcripts, restoring a contiguous and functional host transcript. The ability of group I introns to self-splice and therefore act as ribozymes was first demonstrated by Cech’s group for a group I intron inserted within the nuclear large subunit rRNA gene in the protozoan *Tetrahymena thermophila *[1]. At the same time Michel [2] recognized that organellar group I introns can fold into conserved secondary structures at the RNA level. These observations, when combined with the work by Cech’s group, led to a better understanding of how group I intron ribozymes promote their splicing from transcripts and the ligation of the adjoining exons [3]. Many group I introns can self-splice *in vitro* without assistance from protein co-factors, although splicing *in vivo* is dependent on, or enhanced by, intron- and/or host-encoded factors [4].

Group I introns can be divided into two general classes, those that encode open reading frames (ORFs) and those that do not. Group I introns with ORFs can function as mobile genetic elements that can move within and between genomes by inserting into cognate alleles that lack intron insertions [5]. Here, intron-encoded ORFs function as so-called homing endonucleases (HEases) that cleave intronless alleles to promote a DNA-based recombination-dependent mobility mechanism referred to as intron homing [5,6]. The first experimental connection between DNA endonucleases and intron mobility stemmed from a detailed analysis of the mtDNA yeast omega (ω) locus [7-9]. Mating of two yeast, one with the ω locus and one without the locus, resulted in a much higher frequency of ω inheritance than would be anticipated from random assortment of alleles. Later characterization showed that intron movement was driven by the homing endonuclease encoded within the intron, generating a double-stranded break in the intronless allele at a position close to where the intron is inserted in the intron-containing allele (the intron insertion site). Similar findings of high frequency inheritance of introns were later found from mixed infections of intron-containing and intron-lacking bacteriophages [10]. It is generally assumed, yet infrequently shown experimentally, that these findings may also apply to organelles and to some degree towards bacterial introns.

**Figure 1:**
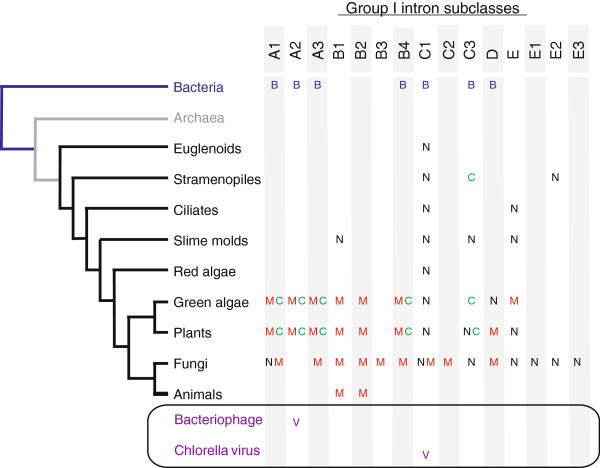
**The distribution and diversity of group I introns.** A small subunit rDNA cladogram shows the biological host range for each group I intron subclass in bacteria (B) and viruses (V). Distribution of group I introns in Eukarya as well as the cellular location of each subclass is indicated (N, nucleus; M, mitochondria; C, chloroplast). This figure was generated based on the available information obtained from the Comparative RNA Website [http://www.rna.icmb.utexas.edu/] and Group I Intron Sequence and Structure Database [http://www.rna.whu.edu.cn/gissd/index.html].

The phylogenomic distribution of group I introns is diverse, as they are found in bacterial, phage, viral, organellar genomes and often nuclear rDNA genes of fungi, plants, and algae (Figure 1). Intriguingly, group I introns are scarce among early branching metazoan mitochondrial genomes [11], and so far have not yet been detected in the Archaea [12]. Bacterial group I introns are mostly confined to structural RNA genes (rRNA and tRNA) and are less frequently inserted within protein-coding genes. Group I introns have also been reported from a variety of bacteriophages [13-15] where they tend to be inserted within conserved protein-coding genes. Other intron and intron-like elements are encountered within prokaryotic genomes, such as group II introns, Archaeal tRNA introns, and bacterial rDNA intervening sequences [16-18], however this review will focus on group I introns.

## Review

### Core features of group I intron RNAs

**Figure 2:**
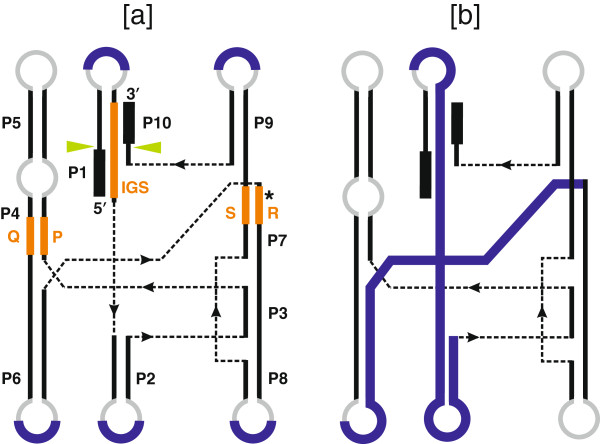
**Secondary structure model for group I introns.** Generic secondary structure representations for group I introns highlighting the locations of intron-encoded proteins. **(a)** The blue lines indicate regions where ORFs that encode homing endonucleases are entirely located in loops. **(b)** In some group I introns, the endonuclease ORFs extend and overlap with intron core sequences. In both panels, stem regions are represented by solid black lines and single-stranded loop regions are represented by grey curved lines. Exon sequences are represented by black boxes. The ten pairing regions (P1 to P10) are also indicated. The solid green arrowheads indicate the intron-exon junctions (5′ and 3′ splicing sites). The positions of the internal guide sequence (IGS) and the so called P, Q, R and S sequence elements are indicated by thick orange lines. The guanosine-5’-triphosphate (GTP) binding pocket within the P7 helix is indicated by an asterisk.

Group I introns are highly variable at the primary sequence level yet possess characteristic conserved secondary and tertiary structures. The secondary structure of group I introns consists of paired (P) elements designated P1 to P10 and single-stranded loop regions (Figure 2). Short, conserved sequences can be recognized in some intron sequences, and these are named P, Q, R, and S. These sequences participate in forming core helical regions, in which as shown in Figure 2 the P sequence pairs with Q (contributing towards the P4 helix) and R pairs with S (contributing towards the P7 helix) [2,19]. The P1 and the P10 helices form the substrate-binding domain wherein the 5′ and 3′ splice sites are juxtaposed to each other [3,20,21]. In some group I introns, P2 is absent. The active core of the group I ribozyme is assembled by two helical domains P4/P6 (P4, P5 and P6), which is considered the scaffolding domain, and P3/P9 (P3, P7, P8 and P9) that form the catalytic domain [21-23]. The P3-P7-P9 helix contains the guanosine-5’-triphosphate (GTP) binding pocket and the exogenous GTP docks onto the G-binding site located in P7. Here the 3′–OH of an exogenous GTP is positioned so that it can attack the 5′-3′ phospodiester bond at the 5′ splice site located within the P1 fold. There is considerable evidence that at least one or more divalent metal ions (preferably Mg^+2^) are present at the active site and contribute towards the catalysis of the group I intron [24,25].

**Figure 3:**
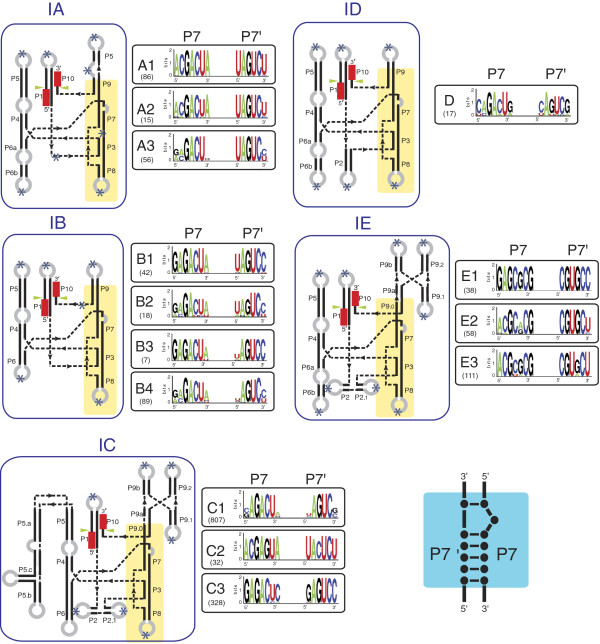
**Differences between group I intron classes (IA to IE).** Shown are secondary structure representatives for the group I intron classes [26-33]. The IA to ID classes are commonly found in bacteria. The IE class is also depicted for comparative purposes. For all group I intron RNA structures the catalytic core is highlighted in yellow. Beside each secondary structure model is a sequence logo alignment of the P7:P7′ pairing for the intron subclasses. The P7:P7′ pairing is important because it is a highly conserved region and is diagnostic for discriminating between various group I intron subclasses. With regards to the sequence logos the information content at each position (in bits, from 0 to 2) is represented by the height of the nucleotide. A score of 2 bits corresponds to high conservation, while a score of 0 corresponds to low conservation. The number of sequences used to generate each sequence logo is indicated below the intron subtype. Asterisks indicate the possible locations of peripheral insertions within the intron. The catalytic domain is highlighted in yellow.

Group I introns have been categorized into five classes, IA, IB, IC, ID and IE [26-28] based on conservation of core domains, alternative configurations of secondary structure elements, the presence of peripheral elements and features of the P7:P7′ helix (for example, P2, P7.1, P7.2) (see Figure 3). Each class is further subdivided based on the presence or absence of specific structural features (that is IA1, IA2 and IA3) [28]. Overall, 14 subgroups of introns have been recognized to date based on structural features [29], and over 20,000 group I introns have been identified or predicted in a variety of organisms. The secondary structures of some group I introns and a list of rDNA intron insertions sites have been compiled in the Comparative RNA Web Site [http://www.rna.ccbb.utexas.edu/] [30], and the group I intron sequence and structure database [28]. Among bacterial group I introns so far, representatives of the following intron subgroups have been noted: IA1, IA2, IA3, IB4, IC1, IC3, and ID [31-33]. When ORFs are present, they are usually entirely inserted in loops that protrude from the core secondary structure (see Figure 2) where the extra sequence associated with the ORF will not interfere with folding of the ribozyme core [34]. In cases where the intron ORF sequence extends into core intron sequences, expression of the intron ORF is tightly controlled so as not to interfere with intron folding and splicing [35,36].

### The mechanism of group I intron splicing

Group I introns are removed from precursor RNA by an autocatalytic RNA splicing event that is mediated by the intron’s RNA tertiary structure. Base-pairing interactions between the 5′-end of the intron and flanking exon sequences define the location of the 5′ and 3′ splice sites. The Internal Guide Sequence (IGS), which is a short intronic sequence near the 5′-end that pairs with sequences of the upstream exon to form P1, determines the 5′ splice site (Figure 2). The 3′ splice site is determined by pairing of a short sequence of the downstream exon with a portion of the IGS, forming P10 and mediating interactions between P9 and the P3/P8 helices that form the catalytic core [3,26,37-39].

**Figure 4:**
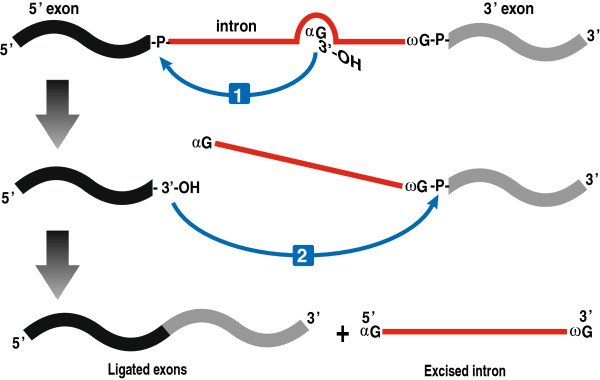
**Schematic representation of group I intron splicing.** The splicing pathway consists of two sequential transesterification reactions. The first reaction is initiated by the 3′–OH group of an exogenous GTP (αG) that docks into the G-binding pocket located in the P7 region and the 3′–OH group attacks the 5′ splice site. In the second reaction, the 3′–OH of the released 5′ exon attacks the phosphodiester bond between the intronic terminal G (ωG) and the 3′ exon, resulting in the liberation of the intron and the ligation of the exons.

Splicing of the group I intron RNA is by a two-step transesterification reaction with an exogenous GTP (αG) with its 3′–OH acting as an initiating nucleophile (Figure 4). Binding of the αG in the G-binding site in P7 positions the 3′–OH of GTP to attack the 5′ splice site. During the first transesterification step the αG is attached to the 5′-end of the intron RNA by a 3′-5′ phosphodiester bond. This step is followed by conformational changes allowing the upstream exon′s terminal 3′ guanosine (ωG) to trade position with the αG and occupy the G-binding site to initiate the second transesterification reaction [26]. The 3′–OH of the upstream exon attacks the 3′ splice site (an interaction facilitated by the formation of P10) promoting the ligation of upstream and downstream exons and the release of the intron RNA [3,40-42]. Splicing is absolutely dependent on a divalent metal ion to stabilize RNA secondary and tertiary structures and to activate the nucleophilic attack by the 3′–OH groups [24,25]. Crystal structures of several group I introns have been resolved, including *Azoarcus* sp. BH72 pre-tRNA^Ile^ intron-exon complexes [24,42,43], *Tetrahymena* pre-rRNA apo enzyme [44,45], and the bacteriophage Twort pre-mRNA ribozyme-product complex [22]. The crystal structures of these introns support the involvement of a two-metal ion mechanism in group I intron splicing.

### Intron- and host-encoded factors that facilitate splicing

Efficient *in vivo* splicing of group I introns often requires proteins with maturase function that can either be intron- or host-encoded [46-50]. The reliance on intron-encoded maturases or host factors implies that the intrinsic intron splicing rate may not be sufficient in a cellular context, and that introns have co-opted cellular factors to facilitate splicing to ensure little or no phenotypic effect on host gene function. For example, three nuclear mutations (*cyt-4*, *cyt-18*, *cyt-19*) were identified that showed cytochrome deficiencies due to defective splicing of the mL2449 group I intron in *Neurospora crassa *[51-53]. Cyt-4 was shown to be an RNase II-like protein that might be involved in the turnover of the excised group I intron [52], and Cyt-18 was revealed to be a tyrosyl-tRNA synthetase that promotes splicing by helping the intron RNA fold into a catalytically active structure [54,55]. Cyt-19 is a member of the DEAD-box protein superfamily of RNA helicases that appears to be an ATP-dependent RNA chaperone that can recognize and destabilize non-native RNA folds that might arise during Cyt-18 mediated folding of group I intron RNAs [29,56-59]. A general theme that emerges from these studies is that intron RNAs interact with cellular RNA-binding proteins to promote the formation of splicing-competent RNA structures.

With regard to bacterial group I introns, comparatively little is known about host- and intron-encoded splicing co-factors [46,49,50]. In the hyperthermophile *Thermotoga neapolitana*, the group I intron interrupting the 23S gene encodes a LAGLIDADG protein with maturase-like activity that stabilizes and activates its cognate intron at high temperatures [47]. Studies on *Escherichia coli* phage T4 introns revealed that host factors such as the StpA protein can act as an RNA chaperone and thus compensate for a group I intron splicing defect *in vivo *[46,60,61]. Ribosomal protein S12 was shown to facilitate the *in vitro* splicing of T4 introns [62], and translation initiation factor IF1 has RNA chaperone activity that can promote the splicing of the T4 phage thymidylate synthase intron [63]. *In vitro* work has shown that eukaryotic proteins such as Cyt-18 [29,64], and DEAD-box proteins like Cyt-19, and Mss116p [59] promote splicing of some bacterial introns, suggesting that bacterial group I introns may benefit from interactions with proteins that assist in intron RNAs folding into splicing competent structures. There is also considerable evidence that the ribosome acts as an RNA chaperone for the T4 introns by sequestering upstream exon sequences that may otherwise compete with intron sequence to form non-productive RNA structures for splicing [65,66]. Collectively, these observations also suggest that intron splicing and gene expression have to be coordinated and therefore introns may not be neutral with regards to their impact on their host cells [36,66].

### Intron-encoded HEases

**Figure 5:**
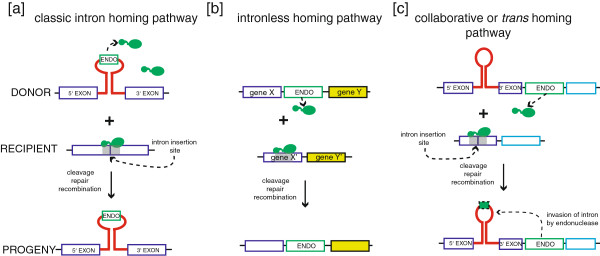
**Mobility pathways mediated by homing endonucleases.** Schematics of different endonuclease-mediated mobility pathways between donor and recipient alleles. **(a)** group I intron homing mediated by intron-encoded endonucleases; **(b)** the collaborative or *trans* homing pathway; **(c)** the intronless homing pathway mediated by free-standing endonucleases. In all cases, the homing endonuclease gene is represented by a green rectangle, and the homing site of the endonuclease is shown by a grey filled rectangle. The green rectangle outlined with dashed line indicates the outcome of a recombination event whereby the endonuclease ORF becomes embedded within an endonuclease-lacking intron, creating a potential mobile group I intron.

Intron-encoded HEases are site-specific DNA endonucleases that recognize and cleave specific target sites (the homing site) in genomes that lack the intron (Figure 5a) [10,67]. Homing sites are typically centered on the intron-insertion site, and include DNA sequences both up- and down-stream of the insertion site (that is in the up- and down-stream exons). The presence of a group I intron thus disrupts the homing site, rendering intron-containing alleles immune to cleavage by their encoded homing endonuclease, and providing a mechanism to discriminate self (intron-containing) from non-self (intronless) alleles. Most characterized HEases possess lengthy recognition sites (> 14 bp) that often encode codons specifying functionally critical amino acids or RNA sequences of the target gene [68-70]. Targeting of conserved sequences is one strategy to ensure that an appropriate homing site is present within closely related genomes. Moreover, many characterized HEases tolerate nucleotide substitutions within their homing sites, facilitating cleavage of variant cognate homing sites that arise by genetic drift.

Currently, there are six families of HEases, classified primarily on the basis of conserved amino acids that correspond to structural or active site residues; the LAGLIDADG, H-N-H, His-Cys box, GIY-YIG, PD-(D/E)xK, and EDxHD families [71-73]. The active site architecture of the His-Cys box and H-N-H families is very similar, and it has been suggested that they are divergent members of a ββα-metal motif. A similar argument can be made for a shared active site architecture of the PD-(D/E)xK and EDxHD families. The LAGLIDADG family is the largest and most diverse group with a wide host range including the organellar genomes of plants, fungi, protists, early branching metazoans, bacterial and archaeal genomes. The GIY-YIG, H-N-H, PD-(D/E)xK, and EDxHD enzymes are most often encoded within group I introns found in phage genomes, and less frequently in introns interrupting genes on bacterial chromosomes. His-Cys box enzymes have an extremely limited phylogenetic distribution, found almost exclusively in protists.

### Intron mobility

Group I intron mobility is catalyzed by the intron-encoded HEases [6,74,75] (Figure 5). The HEases have specific target sites, with some allowance for sequence variation in their homing sites (Figure 5a). Recognition of variant homing sites ensures propagation in the face of substitutions that accumulate over time in the target site. Recently, *trans*-acting HEases have been described in T4 and related phages that can promote the homing of either group I introns lacking ORFs or group I introns that encode defunct (degenerated) HEases (Figure 5b) [67,72,76,77]. Intron homing is initiated by the HEase that introduces a double-strand break (DSB), or nick, in an intronless allele [77]. The homing process is completed by host DSB-repair or synthesis-dependent strand annealing (SDSA) pathway [78-81] that use the intron-containing allele as a donor to repair the break in the recipient intronless allele (Figure 5). The end result is the nonreciprocal transfer of the mobile intron element into the intronless allele (that is recipient). As stated previously, nicking HEases can stimulate intron mobility but the actual mechanism of how a single-strand nick stimulates recombination is not understood. The homing event is frequently associated with co-conversion of markers flanking the intron insertion site, and the HEase can influence the extent of co-conversion by remaining bound to one of the cleavage products, preventing access of the recombination and repair machinery including exonucleases [79,80,82,83]. It should be noted that homing endonuclease genes can be free-standing and move into new sites by a mechanism referred to as intronless homing, a mechanism that is similar to the one described above (see Figure 5c).

It is generally thought that group I introns propagate through a population of intronless alleles with ‘super-Mendelian’ inheritance, and that all available alleles for homing quickly become occupied. At this point, the HEase can quickly accumulate deleterious mutations that inactivate the enzyme, or the HEase assumes another function (possibly a maturase) to avoid loss. Alternatively, it is thought that group I introns can ‘escape’ to a new population of intronless alleles by transposition to new sites (ectopic integration) by reverse splicing. Reverse splicing is the reverse of the forward splicing reaction, and theoretically allows a group I intron RNA to insert into a RNA molecule with four to six complementary bases to the P1 stem of the intron RNA [84,85]. This proposed pathway of RNA-based mobility also requires the additional steps of reverse transcription of the reverse-spliced intron and target RNA followed by integration of the cDNA into the genome by recombination, yet there is no direct experimental evidence to support this pathway. The best circumstantial evidence for reverse splicing has been documented for rDNA introns where related introns are inserted in two different locations within rDNA genes [55,86].

Another mechanism for ectopic integration or transposition relates to the relaxed specificity of many intron-encoded HEases. For instance, cleavage at a site similar to a HEase’s native target site may promote intron mobility, and it has been shown that the cleavage specificity of the I-TevI HEase can be influenced by oxidative stress [87]. However, the low cleavage rates at ectopic sites will limit the frequency of intron movement by this mechanism. Because homologous recombination between unrelated sequences will be inefficient, it is thought that illegitimate recombination pathways would be necessary for intron transposition [88].

### Domestication of group I introns and the formation of novel genetic elements

There are a few instances where group I introns or their components may have been domesticated by their host genomes, or by other types of mobile genetic elements. The bacterial DUF199/WhiA protein is a transcription factor and its N-terminal region contains the same protein fold as found in monomeric LAGLIDADG HEases encoded within group I introns [89,90]. This similarity suggests that an invasive element was co-opted to serve as a regulatory protein [91]. The ability of group I intron RNAs to form complex tertiary structures has been harnessed in *Clostridium difficile* as a feature of a two-component riboswitch that involves c-di-GMP as an allosteric activator [92]. Here, in the 5′ untranslated region of an mRNA, a c-di-GMP binding aptamer is located upstream of a group I intron; the binding of c-di-GMP to its aptamer modifies the group I intron fold and shifts the 5′ splice site. In the presence of c-di-GMP, RNA processing yields an mRNA where the ribosome binding site is moved upstream of the start codon, whereas splicing without c-di-GMP results in a version of the transcript where the ribosome binding site is removed as part of the intron RNA [92]. In essence, the allosteric self-splicing intron has been domesticated as a metabolite sensor and genetic regulatory element.

A unique composite element has been described in some enterotoxin producing strains of *C. difficile* in the *tcdA* locus. The composite element, termed an IStron, is composed of a splicing-competent group I intron (IA2 subgroup) that has an insertion element (IS, of the IS605 element family) embedded within its 3′-end and encoding two transposases [93,94]. One of the transposases is a TnpA-like protein that belongs to the HUH endonuclease superfamily [95]. TnpA can promote mobility events of the IS200/IS605 family of bacterial insertion elements by cleavage and rejoining of single-stranded DNA. These endonucleases cleave their target sites by cutting the lagging strand within a DNA replication fork [96,97]. This mobility mechanism might be analogous to how the H-N-H family of nicking HEases promotes the mobility of group I introns. IStrons have the potential to transpose into genes but its capacity to self-splice should minimize its impact on the host gene [98]. Although IStrons appear to have the best of both worlds in the sense that they encode elements to promote spread (transposase) and aid in their persistence (self-splicing intron), they have limited phylogenetic distribution [99,100].

### Group I intron distribution in bacteria: genes and genomes

Within bacteria, group I introns are predominately inserted within structural RNA genes such as tRNA and rRNA genes [31-33,101-107]. This bias has been explained in part by the conservation among structural RNA genes. Conversely, insertion of group I introns into protein-coding genes may be selected against, as the coupling of transcription and translation would interfere with folding of the group I intron to facilitate ribozyme formation and thus splicing [13,108]. The presence of a stop codon in-frame with the upstream exon of many group I introns is viewed as evidence that stalling of the ribosome might be a strategy to facilitate intron RNA folding and splicing [98,108-110]. Nevertheless, there have been reports of bacterial protein-coding genes that have been invaded by group I introns, such as the flagellin gene in a thermophilic *Bacillus* species [111,112], *recA* and *nrdE* genes in various *Bacillus* species [99,113], and some cyanobacterial *nrdE* genes [109,110]. This trend of insertion into protein-coding genes is particularly evident in bacteriophages, as all introns observed to date are inserted in protein-coding genes, in spite of the presence of many phage-encoded tRNA genes [14,100,114-117]. This distribution may be related to the fact that optimal DNA targets for HEases occur within conserved protein-coding genes, which, in the context of the relatively small coding potential of many phage genomes, includes targets such as DNA polymerases, ribonucleotide reductases, and terminases.

Interestingly, group I introns have so far not been discovered in archaeal genomes, although group I intron derived HEase sequences are sometimes associated with archaeal introns [117-122]. The archaeal-specific introns are removed by a mechanism that involves tRNA splicing endonucleases [12,123-126]. It has been suggested that the efficient protein-dependent splicing of archaeal introns may have outcompeted RNA-based self-splicing introns by minimizing any phenotypic effect on host genomes from slow *in vivo* splicing rates, and that self-splicing RNA introns became extinct in the archaeal lineage [12]. This scenario implies a cost associated to the host genome with maintaining group I ribozyme based splicing elements and/or their co-factors (maturases/chaperones), which may have limited their spread and persistence of self-splicing introns among the bacteria and their associated phages.

The persistence and spread of group I introns in prokaryotic genomes is dependent on a number of factors including (1) the phenotypic cost associated with the insertion of a group I intron, (2) the availability of intronless alleles for endonuclease-mediated homing, (3) the presence of efficient homology-based DSB repair systems, (4) the availability of DNA or RNA transfer mechanisms such as DNA uptake by natural transformation, conjugation and plasmid transfer, and phages. Interestingly, recent work on the *Bacillus cereus* group suggested that some of the genomic *recA*, *nrdE*, *nrdF* introns are similar to phage introns, indicating that phage infection could serve as a vector system for the lateral movement of introns among different genomes [100]. However, there is little evidence to show that bacterial introns are moved horizontally among bacterial species. One study [127] showed that placing a group I intron from *Tetrahymena* into the *E. coli* 23S gene resulted in the reduction of the growth rate which was correlated with poor splicing of the *Tetrahymena* intron. Moreover, the intron RNA was shown to associate with the 50 S ribosomal subunit and possibly interfere with translation. Clearly, there are barriers to intron spread in bacteria [13] that are curiously absent from organellar genomes where group I introns are very abundant.

### The evolution of a composite mobile element

One of the most intriguing questions about mobile group I introns concerns their evolutionary origin. The current consensus is that HEases and group I introns had distinct evolutionary origins, and that HEases have on multiple independent occasions invaded an endonuclease-free intron. The alternative scenario, that group I introns always possessed an endonuclease gene is problematic for a number of reasons, including the fact that many group I introns do not contain ORFs, and the notion that group I introns were direct descents of catalytic RNAs from the RNA world. Moreover, the finding that HEases can exist outside of the protective confines of introns, as so-called free-standing homing endonucleases, lent credibility to the hypothesis that these free-standing enzymes could be a potential source of the ‘invading’ endonuclease. Two mechanisms that would lead to the formation of such a composite mobile intron have been proposed. Loizos *et al*. [128] noted that in the *sunY* gene of the T4 phage the intron sequences flanking the HEase ORF (I-TevII) were similar to the exon junction sequences that comprise the I-TevII target sequence. Importantly, they were able to demonstrate that a synthetic construct that included the fused sequence composed of the up- and down-stream sequences that flank the I-TevII ORF was indeed cleaved by I-TevII. This result provided strong circumstantial evidence for the ‘endonuclease-gene invasion’ hypothesis whereby a free-standing HEase cut an intron sequence that fortuitously contained a similar HEase target site. During the recombination-based repair process, the endonuclease gene sequence was inserted into the cleaved intron sequence, thus generating a composite potentially mobile intron.

Recent studies [72,76] provide a second mechanism, termed collaborative homing, for the origin of mobile introns. Work on two different phages revealed systems where a free- standing HEase and an ORF-less group I intron converged on the same conserved target site (Figure 5b). That is, the target site of the endonuclease corresponded to the intron-insertion site. Thus, the endonuclease was ‘pre-adapted’ to target the intron-insertion site, and an illegitimate recombination event that moved the free-standing endonuclease gene into the intron would quickly create an efficient composite mobile intron capable of mobility [76].

Regardless of the origin of mobile group I introns, one would assume that endonuclease invasion would have a deleterious effect on intron splicing. In this respect, it is interesting to note that many endonuclease ORFs are inserted in loops that presumably do not interfere with folding and splicing. It is also possible that the intron-encoded endonucleases and/or host factors were able to compensate by stabilizing the intron tertiary RNA structure or discouraging misfolding of the intron RNAs [129-132]. This would effectively stabilize the intron/endonuclease relationship within the genome as splicing competency would be under a strong selective pressure if the intron was inserted in a functionally important gene. Long-term persistence of the composite element is dependent on the opportunity to invade intronless alleles, as detailed by Goddard and Burt and others [132,133].

This returns us to the enigma of why group I introns and their associated HEases have been successful in spreading among the organellar genomes of plants, protozoans, and fungi but have very limited representation among bacterial and phage genomes. Koonin [134] proposed that group I introns evolved as parasitic selfish-RNAs (ribozymes) in abiotic compartments that housed early forms of the ‘RNA world’. If indeed these elements are ancient, it is surprising that now they have such a limited distribution, being absent in the Archaea and only rarely encountered among bacteria. One intriguing possibility is that the CRISPR/Cas RNA-based genome defense system, that restricts foreign DNAs such as plasmids or phage DNAs, has a role in limiting the spread of mobile group I introns present on these elements, specifically the type III CRISPR systems can target ssRNA in addition to DNA [135-137]. An interesting observation is that CRISPR/Cas systems are extremely prevalent in Archaea, but less so in bacteria, correlating with the absence of group I introns from Archaea.

## Conclusions

The mechanisms that promote and prevent group I introns from proliferating among bacterial genomes are poorly understood, as is the long-term impact of introns on organismal viability. When present, it is assumed that introns are phenotypically neutral, yet the co-opting of intron functions by a riboswitch or the domestication of intron-encoded homing endonuclease as a regulatory protein (WhiA) indicates that introns can be a source of genetic novelty. Future research efforts directed at understanding the effect of group I introns on host gene expression, mechanisms of mobility to ectopic sites and their spread among bacterial genomes and phages will lead to valuable insights regarding the dynamics and evolution of group I introns.

## Abbreviations

bp: base pair; c-di-GMP: cyclic diguanylate; DSB: double-strand break; GTP: guanosine-5′-triphosphate; HEase: homing endonuclease; HEG: homing endonuclease gene; HUH: endonuclease motif; IGS: Internal Guide Sequence; IS: insertion element; ORF: open-reading frame; rRNA: ribosomal RNA tRNA, transfer RNA; SDSA: synthesis-dependent strand annealing.

## Competing interests

The authors declare that they have no competing interests.

## Author’s contributions

GH: conception and design, figure preparation, manuscript writing and final approval of manuscript. MH: conception and design, compilation of data for Figures 1, 2 and 3, final approval of manuscript. DRE: conception and design, figure preparation, manuscript writing and final approval of manuscript. All authors read and approved the final manuscript.
